# Linkage disequilibrium across two different single-nucleotide polymorphism genome scans

**DOI:** 10.1186/1471-2156-6-S1-S86

**Published:** 2005-12-30

**Authors:** Juan Manuel Peralta, Thomas D Dyer, Diane M Warren, John Blangero, Laura Almasy

**Affiliations:** 1 Centro de Investigación en Biología Celular y Molecular, Ciudad de la Investigación, Universidad de Costa Rica, San José, Costa Rica; 2 Genomics Computing Center, Department of Genetics, Southwest Foundation for Biomedical Research, San Antonio, TX 78227-5301, USA

## Abstract

Linkage disequilibrium (LD) content was calculated for the Genetic Analysis Workshop 14 Affymetrix and Illumina single-nucleotide polymorphism (SNP) genome scans of the Collaborative Study on the Genetics of Alcoholism samples. Pair-wise LD was measured as both D' and *r*^2 ^on 505 pedigree founder individuals. The *r*^2 ^estimates were then used to correct the multipoint identity by descent matrix (MIBD) calculation to account for LD and LOD scores on chromosomes 3 and 18 were calculated for COGA's ttdt3 electrophysiological trait using those MIBDs. Extensive LD was observed throughout both marker sets, and it was higher in Affymetrix's more dense SNP map. However, SNP density did not solely account for Affymetrix's higher LD. MIBD estimation procedures assume linkage equilibrium to construct genotypes of non-genotyped pedigree founder individuals, and dense SNP genotyping maps are likely to contain moderate to high LD between markers. LOD score plots calculated after correction for LD followed the same general pattern as uncorrected ones. Since in our study almost half of the pedigree founders were genotyped, it is possible that LD had a minor impact on the LOD scores. Caution should probably be taken when using high density SNP maps when many non-genotyped founders are present in the study pedigrees.

## Background

Single nucleotide polymorphisms (SNPs) are ubiquitous throughout the human genome. SNPs are more closely spaced than microsatellites and they permit the construction of very high-density genome screening maps. Methods that rely on SNPs are very easy to automate (no electrophoresis, easy allele calling), and will probably be more cost effective than microsatellites for performance high-throughput genotyping.

Yet, several properties of SNP maps remain unclear. Kruglyak [1] evaluated the usefulness of diallelic markers for linkage analysis by evaluating how many of them would be needed to extract the same amount of genetic information as microsatellites. With the help of simulation, he concludes that a 1–2 cM diallelic map will be equivalent or even superior to the conventional 5–10 cM microsatellite map, even in cases in which allele frequencies of the diallelic markers are far from the ideal 50/50 ratio. More recently, Goddard and Wijsman [2] pointed out the relevance of marker information, map accuracy, and flexibility of analysis as important factors to consider for diallelic maps. They argue in favor of clustered SNP maps, with 2–3 SNPs per cluster.

The impact of linkage disequilibrium (LD) on linkage studies has also been the subject of some discussion [3] and analysis [4], and this issue still raises several questions. For instance, the estimation of multipoint identity-by-descent (MIBD) matrices generally assumes that haplotypes for non-genotyped founders can be imputed using the product of the marker allele frequencies involved, effectively implying that the haplotype markers are in linkage equilibrium (LE). Because SNPs are much more closely spaced than traditional microsatellites, the assumption that the markers are in LE can be easily violated. The effect that such departures from LE will have on the LOD score statistic is yet unclear.

Affymetrix and Illumina SNP genotyping of samples from the Collaborative Study on the Genetics of Alcoholism (COGA) for the Genetic Analysis Workshop 14 (GAW14) provide an excellent opportunity to investigate certain properties of SNP genome screening maps. It also leads to the comparison of several aspects of both company products, one of the most obvious being the performance of the highly dense Affymetrix in comparison with the more sparse Illumina array.

Here, our main objective is to explore empirically the extent of LD that is present in the two complete SNP genome scans of real DNA samples and its effect on the LOD score.

## Methods

SNP genotypes from the clean GAW14 COGA datasets were selected from Affymetrix's GeneChip Mapping 10 K Array (AMA) and Illumina's Linkage Panel III (ILP). A total of 11,120 Affymetrix and 4,720 Illumina SNP markers (SNPs) were present in the final clean datasets, but only 10,084 and 4,599 of those SNPs, respectively, were used for this analysis. One thousand and thirty-six Affymetrix and 121 Illumina markers were not included in the first dataset that was distributed for GAW 14. Those markers became available after a substantial part of the work presented here was done and therefore were not included in the analysis. COGA pedigree founders (*n *= 505; 240 genotyped, 265 not genotyped) were chosen as a representative sample of unrelated individuals, and only their genotypes were included in the LD analysis.

LD was measured pairwise as D' and *r*^2 ^[5]. Pairs were composed of SNP markers from the same chromosome and dataset, and all consecutive adjacent marker pairs were constructed for the 22 autosomes. Pairs constructed for AMA and ILP SNPs will be referred as AMASnP and ILPSnP, respectively. The expectation-maximization (EM) method implemented in GENECOUNTING [6] was used to obtain maximum-likelihood frequency estimates of the required two-marker haplotypes. As pointed out by Schaid et al. [3], the EM algorithm does not assume LE between markers. Sex chromosomes were not included in the analysis. To assess the degree of spurious LD present in the sample, unlinked markers from chromosomes 1 and 2 were combined systematically in pairs, starting at 0 cM and moving towards the opposite telomere. LD between them was measured as described above.

Polymorphism information content (PIC) [7-9] was also calculated for each SNP marker and each two-SNP marker haplotype (treated as a single 4-allele marker). All LD and PIC measures were assigned to the average genetic distance between the markers forming each pair.

For AMA and ILP SNP markers on chromosomes 3 and 18, MIBD matrices were calculated using LOKI [10]. Chromosomes 18 and 3 were selected because they showed candidate regions with LOD scores ≥ 3 for COGA's ttdt3 electrophysiological trait phenotype. These MIBDs were a mixture of individual SNP markers and SNP markers combined into haplotypes from pairs of markers that showed LD (measured as *r*^2 ^as described previously) above three arbitrary thresholds: 0.2, 0.4, and 0.6. A total of 12 (2 SNP sets*2 chromosomes*3 thresholds) "corrected" MIBDs were constructed as described, using all genotyped individuals (not only founders). Four (2 SNP sets*2 chromosomes) additional "uncorrected" MIBDs were used as the comparison standard for the LOD scores. Multipoint LOD scores were then calculated for the ttdt3 trait with SOLAR [11], using the methods described by Warren et al. [12], with each of the MIBDs.

Reported *p*-values are uncorrected for multiple testing.

## Results

Overall, pair-wise haplotype frequencies were estimated for a median of 488 Illumina (range = 486–520) and 490 Affymetrix (range = 276–520) SNP haplotypes per chromosome. Nineteen AMA SNPs had to be discarded from the analysis because they were not polymorphic in the sample. Except for chromosomes 19 and 22 AMA had a more dense marker coverage of each chromosome.

Random LD between unlinked markers was found to be low, for both *r*^2 ^and D', in ILP (mean *r*^2 ^= 4.35 ± 0.34 × 10^-3^, mean D' = -2.45 ± 5.22 × 10^-3^) as well as in AMA (mean *r*^2 ^= 5.51 ± 0.29 × 10^-3^, mean D' = -7.44 ± 10.09 × 10^-3^). But variance was greater in AMA's *r*^2 ^and D' (F_749,380 _= 1.43, *p *<< 0.0001 and F_749,380 _= 7.35, *p *<< 0.0001, respectively). Only 0.26% (*N *= 380) ILPSnP showed |D'| ≥ 0.5, while 6.85% (*n *= 759) AMASnP did. All ILPSnP and AMASnP *r*^2 ^values for unlinked markers were below 0.06.

We analyzed 10,065 AMA and 4,598 ILP linked SNP pairs. Of all AMASnP, 8,096 (80.44%) were pairs in which the distance between the SNPs was ≤ 1 cM, while only 1,242 (27.01%) ILPSnP were formed by markers at that spacing. The mean distance between the two SNPs in a pair was significantly different (T_10064,4597 _= 24.56, *p *< 0.0001) between AMA (3.08 ± 0.07 × 10^-1 ^cM), and ILP (7.48 ± 0.16 × 10^-1 ^cM) pairs. Variance in distance between AMASnP SNPs was significantly lower (F_10064,4597 _= 0.44, *p *< 0.0001) than between ILPSnP SNPs.

**Figure 1:**
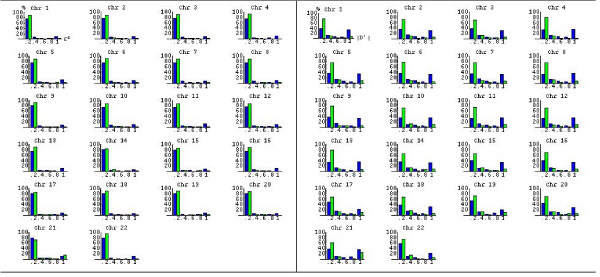
**LD frequency across Affymetrix and Illumina SNP maps**. LD frequency distribution of Affymetrix (blue/black bars) and Illumina (green/gray bars) SNP maps, as measured by *r*^2 ^(left) and |D'| (right). Bar names are the LD category boundaries. Note "U" shaped distribution of the LD content of Affymetrix map, and the general increase of LD, when measured as |D'|.

Figure 1 shows AMASnP and ILPSnP *r*^2 ^and D' LD frequency plots for all 22 chromosomes. The extent of LD, measured either as *r*^2 ^or D', is much more conspicuous between AMASnP. Most of the observed *r*^2 ^values are ≤ 0.2 for both SNP sets, but |D'| values show maximum frequencies at values ≤ 0.2 and >0.8 only for Affymetrix SNPs. At a SNP spacing within a pair ≤ 1 cM, 3,241 (40.03%) of all AMASnP had measures of |D'| ≥ 0.5, while only 37 (2.98%) of all ILPSnP did. At the same ≤ 1 cM spacing, 960 (11.87%) AMASnP and 8 (0.64%) ILPSnP pairs had an *r*^2 ^≥ 0.5 measure.

Mean PIC values were found to be significantly lower (T_10064,4597 _= 90.18, *p *<< 0.0001) for AMA (0.2855 ± 0.0006) than for ILP (0.3536 ± 0.0003) SNPs. PIC was also significantly lower (T_10064,4597 _= 85.56, *p *< 0.0001) for AMA (4.92 ± 0.01 × 10^-1^) than for ILP (6.41 ± 0.01 × 10^-1^) haplotypes. Total PIC variance was significantly higher in AMA than in ILP for both SNPs (F_10064,4597 _= 8.28, *p *<< 0.0001) and haplotypes (F_10064,4597 _= 3.21, *p *<< 0.0001).

**Figure 2:**
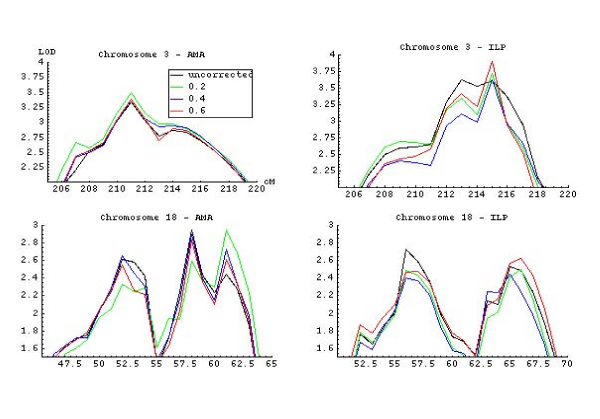
**LOD scores obtained using MIBDs uncorrected and corrected for LD**. These are plots of the maximum LOD score region of chromosomes 3 and 18 of the COGA ttdt3 trait. LOD scores were calculated using MIBDs constructed by removing SNP pairs that in founders showed LD as *r*^2 ^≥ {0.2, 0.4, 0.6}. LOD scores calculated using uncorrected MIBDs are also shown.

Using AMA uncorrected MIBDs, the ttdt3 trait phenotype maximum LOD peaks were 2.94 at 58 cM from 18*p*-ter and 3.34 at 211 cM from 3*p*-ter, while ILP's uncorrected MIBDs gave LOD peaks of 2.73 at 58 cM from 18*p*-ter and 3.63 at 213 cM from 3*p*-ter. Figure 2 shows a plot of the region with the highest LOD scores obtained for the AMA and ILP SNP sets at the different arbitrary *r*^2 ^thresholds used. LOD score curves tended to follow the same general pattern of LODs calculated using the uncorrected MIBDs, with three exceptions that are worth mentioning: a shift from 58 cM to 61 cM (chromosome 18) in the location of the maximum LOD when AMA's 0.2 MIBD was used; and both, a 8-cM shift from 58 cM to 66 cM (chromosome 18) and a shift from 213 cM to 215 cM (chromosome 3) when ILP's 0.2, 0.4, and 0.6 MIBDs were used.

## Discussion

As both the AMA and ILP genome scans show, extensive LD is present throughout the genome. Yet it is more extensive within AMA haplotypes, something which *per se *is not surprising, given that AMA's higher marker density causes SNPs to be more closely spaced. What's striking is that for markers under the same < 1-cM marker spacing, AMA shows a much higher proportion of LD than ILP (~40% vs ~3% respectively, see Results). This suggests that there might be reasons, other than marker spacing, that affect LD content. For instance, Affymetrix and Illumina certainly have different protocols to select the SNPs that are in their AMA and ILP products. Differences in the SNP selection procedure might explain why we observed significantly more LD in the Affymetrix product.

The AMA and ILP genome scans also differ in the quantity of SNP and haplotype information. PIC was much more variable in the AMA dataset, and it was consistently lower for both SNPs and haplotypes. One possible explanation for this is that closely spaced markers became redundant because of the high LD present between them.

What seems to be clear is that LD is a common feature of the genome. Tsunoda et al. [13] focused on 77,176 SNPs, located in 14,271 genes, genotyped for 1,128 chromosomes also found regions of the genome showing extensive LD.

John et al. [4] also recently published an empirical comparison between SNP and microsatellite whole-genome scans. They used Affymetrix GeneChip Mapping 10 K Arrays and found areas of LD. They reported that across a 40-cM region of chromosome 6, 45% of the SNP pairs had an *r*^2 ^> 0.4. John et al. [4] tried to correct the IBD calculations for high LD, using only SNPs that were in LE, and found "modest" changes in linkage results [4]. But because removing SNPs that show LD results decreases the information content present for the linkage, they tried unsuccessfully to compensate for it by replacing them with haplotypes generated by the EM algorithm.

Here, we explored the effect of LD on the LOD score by accounting for the amount of it in the founder individuals, at different stringencies (*r*^2 ^thresholds), when creating the MIBD matrices. We found "modest" LOD score changes in magnitude, as John et al. did. But those modest changes in magnitudes were able to shift the location of the maximum LOD score even by a great genetic distance (10 cM, see Figure 2). Because we do not know the true location of the quantitative trait locus (QTL) affecting COGA's ttdt3 is (or if such a QTL exists), it is impossible for us to determine the relevance that this finding might have for QTL/gene mapping.

In spite of this, the main features of the LOD score curves remain the same, suggesting that the MIBD estimation algorithm used might be robust enough to tolerate the violation of its implicit assumption of LE. Another possible, and maybe more likely, explanation could be that the COGA families analyzed here do not provide a good framework to test for the effect of LD on LODs through MIBD estimation. Because we had genotypes for about half the founders, few founder haplotypes had to be estimated during MIBD construction by means of marker allele frequencies. In effect, that could diminish the impact of LD on the LOD scores by means of fewer violations of the LE assumption. If this is indeed the case, then LD could potentially be a serious problem for studies that have many non-genotyped founder individuals in their pedigrees.

Finally, one more practical issue deserves consideration. These technologies generate vasts amounts of information that will be statistically analyzed. Those statistical analyses have a non-trivial computational cost associated with them. Users of these technologies have to evaluate the wet-laboratory savings they bring in light of the not-so-evident computational and statistical costs that will be needed after data collection. Affymetrix's approach is more costly in this sense, not because it produces a higher volume of information to be analyzed, but because part of it is redundant and time and/or resources need to be allocated for its analysis.

## Conclusion

SNP genotyping technologies are becoming more widespread and allow for very high density (less than a centimorgan) whole-genome scans. But as marker map density increases, so does LD content. We showed that considerable LD exists between markers in both the Affymetrix and Ilumina SNP genotyping sets, and it is more pronounced in Affymetrix's denser map. Since all methods used to calculate MIBDs assume LE when estimating haplotypes of non-typed founder individuals, the effect of violating this assumption using highly dense SNP maps in which LD is more the rule than the exception needs to be considered. We observed modest changes in LOD score magnitude and shifts in the position of the maximum LOD after correcting for LD in the MIBDs. But the effect of LD on LOD scores might not always be this subtle and it may be adverse in studies where a large number of founder individuals are not genotyped.

## Abbreviations

AMA: Affymetrix GeneChip Mapping 10 K Array

COGA: Collaborative Study of the Genetics of Alcoholism

EM: Expectation maximization

GAW14: Genetic Analysis Workshop 14

ILP: Illumina Linkage Panel III

LD: Linkage disequilibrium

LE: Linkage equilibrium

MIBD: Multipoint identity by descent

QTL: Quantitative trait locus

PIC: Polymorphism information content

SNP: Single-nucleotide polymorphism

## Authors' contributions

JMP performed the statistical analysis and drafted the manuscript. TD did the data cleanup and helped with the preparation of the MIBD matrices. LA designed the study, participated in its coordination, and helped to draft the manuscript. DMW selected the chromosomes, the trait, and the model for the linkage analysis. JB participated in the design of the study.
